# Interventions to Improve Movement and Functional Outcomes in Adult Stroke Rehabilitation: Review and Evidence Summary

**DOI:** 10.2196/jopm.8929

**Published:** 2018-01-18

**Authors:** Susan Hamady Lin, Timothy P Dionne

**Affiliations:** ^1^ Institute of Health Professions Department of Occupational Therapy Massachusetts General Hospital Institute of Health Professions Boston, MA United States; ^2^ Department of Rehabilitation Sciences School of Public Health and Health Professions University at Buffalo, State University of New York Buffalo, NY United States

**Keywords:** stroke, evidence-based health care, patient-centered care, review

## Abstract

**Background:**

Patients who have had a stroke may not be familiar with the terminology nor have the resources to efficiently search for evidence-based rehabilitation therapies to restore movement and functional outcomes. Recognizing that a thorough systematic review on this topic is beyond the scope of this article, we conducted a rapid review evidence summary to determine the level of evidence for common rehabilitation interventions to improve movement/motor and functional outcomes in adults who have had a stroke.

**Objective:**

The objective of this study was to find evidence for common rehabilitation interventions to improve movement/motor and functional outcomes in adults who have had a stroke.

**Methods:**

Medline Complete, PubMed, CINAHL Complete, Cochrane Database, Rehabilitation and Sports Medicine Source, Dissertation Abstracts International, and National Guideline Clearinghouse, from 1996 to April of 2016, were searched. From 348 articles, 173 met the following inclusion criteria: (1) published systematic reviews or meta-analyses, (2) outcomes target functional movement or motor skills of the upper and lower limbs, (3) non-pharmacological interventions that are commonly delivered to post-stroke population (acute and chronic), (4) human studies, and (5) English. Evidence tables were created to analyze the findings of systematic reviews and meta-analyses by category of interventions and outcomes.

**Results:**

This rapid review found that the following interventions possess credible evidence to improve functional movement of persons with stroke: cardiorespiratory training, therapeutic exercise (ie, strengthening), task-oriented training (task-specific training), constraint-induced movement therapy (CIMT), mental practice, and mirror therapy. Neuromuscular electrical stimulation (NMES) (ie, functional electrical stimulation) shows promise as an intervention for stroke survivors.

**Conclusions:**

Most commonly delivered therapeutic interventions to improve motor recovery after a stroke possess moderate quality evidence and are effective. Future research recommendations, such as optimal timing and dosage, would help rehabilitation professionals tailor interventions to achieve the best outcomes for stroke survivors.

## Introduction

While the mortality rate from stroke has declined by 35% from 2001 to 2011, stroke remains the leading preventable cause of disability, leaving many stroke survivors with daily challenges [1], such as impairments in mobility, activities of daily living, house maintenance tasks, leisure activities, and stamina [2]. Stroke rehabilitation interventions are therefore critically important to maximize functional recovery and independence. Although an evidence summary of exercise therapy was published in 2005, it was not specific to stroke and was written in Dutch [3].

Among the many stakeholders interested in outcomes, clinicians and patients/caregivers seek trustworthy information about therapies (ie, evidence summary). Clinicians and policy-makers may not have the time to comprehensively research, given the rapid proliferation of research [4]. As patient-centeredness is increasingly adopted in clinical settings, it is important to not only respect patients’ preferences, but to also facilitate patients’ engagement and knowledge about their health condition [5]. Patients who have had a stroke may not be familiar with the terminology nor have the resources to efficiently search for evidence-based rehabilitation therapies to restore movement and functional outcomes. Recognizing that a thorough systematic review on this topic is beyond the scope of this article, we conducted a rapid review evidence summary to determine the level of evidence for common rehabilitation interventions to improve movement/motor and functional outcomes in adults who have had a stroke.

## Methods

Rapid review evidence summaries provide trustworthy information for broad questions to end users in a timely manner [6]. We specified the following inclusion criteria: (1) published systematic reviews and meta-analyses, (2) outcomes include functional movement or motor skills of the upper and lower limbs, (3) non-pharmacological interventions commonly delivered to poststroke population (acute and chronic), (4) human studies, and (5) English language. We excluded interventions that are not commonly delivered in postacute care settings, such as aquatic therapy and robotics. We sought Level 1 evidence from Medline Complete, PubMed, CINAHL Complete, Cochrane Database, Rehabilitation and Sports Medicine Source, Dissertation Abstracts International, and National Guideline Clearinghouse, from 1996 to April of 2016. Search terms included similar terms of the intervention, as well as *stroke*, *systematic review*, and *meta-analysis* (see Table 1) *.* When questions arose about article inclusion or search terms, we discussed these items and rationales until an agreement was reached.

We screened 348 articles and identified 173 articles that met the inclusion criteria (see Table 2). Evidence tables were constructed to categorize and describe the results. After analysis, narrative summaries were written for each category.

**Table 1:** Search terms for interventions.

Category	Search terms used
Exercise–resistance training	Exercise, strength, resistance training, progressive resistance, physical activity, circuit training, cardiorespiratory, exercise therapy, function, intervention, mobility, motor, stroke, systematic review, evidence synthesis, meta-analysis
Constraint induced movement therapy	Constraint-induced, movement, systematic review, meta-analysis, stroke
Task-oriented training	Task-oriented training, task-specific training, stroke, systematic review, meta-analysis, repetitive task practice
Mirror therapy	Mirror therapy, systematic review, meta-analysis, stroke, motor
Neuromuscular electrical stimulation	Electrical stimulation, electrostimulation, electric stimulation, neuromuscular stimulation, systematic review, meta-analysis, stroke, motor
Mental practice	Mental practice, mental rehearsal, motor imagery, systematic review, meta-analysis, stroke

**Table 2:** Search results.

Category	Number of articles	Articles meeting criteria
Exercise–resistance training	165	55
Task-oriented training	54	35
Constraint induced movement therapy	34	26
Mental practice	22	14
Mirror therapy	19	12
Neuromuscular electrical stimulation	54	31
Total	348	173

## Results

### Interventions

In stroke rehabilitation, practitioners can choose among many rehabilitation interventions but this article will focus on interventions that facilitate functional movement and motor outcomes.

Based upon systematic reviews, common motor interventions include: cardiorespiratory training, therapeutic exercise, constraint-induced movement therapy (CIMT), task-oriented training or repetitive task practice, mental practice, mirror therapy, and neuromuscular electrical stimulation [7-9]. Interventions can vary by application, including method, therapist skill and familiarity of intervention, and amount of patient participation [10].

### Cardiorespiratory Training

Cardiorespiratory training and aerobic exercise provide several health benefits to survivors of stroke. Two meta-analyses support aerobic exercise’s positive effect on walking speed and walking endurance [11,12], but these effects do not extend to the Timed Up and Go (TUG) Test [11], Berg Balance score, Functional Independence Measure score [12]. Similarly, systematic reviews reported that gait-oriented cardiorespiratory training improves walking speed and tolerance [13,14], walking distance and peak oxygen uptake [15], and walking capacity [16]. Mixed training resulted in weaker effects on walking, and possibly balance [17].

While one systematic review reported an insufficient level of evidence for cardiovascular exercise’s effects on disability, impairment, extended activities of daily living, and mortality, this review was published in 2003 and only included three trials [18]. Recent systematic reviews have concluded that cardiorespiratory training and exercise improve disability during or after usual stroke care [16,19] and improve health-related quality of life, respectively [20,21]. A review of 58 trials reported that cardiorespiratory training can produce moderate improvement on global indices of disability (standardized mean difference [SMD]=0.52, 95% confidence interval [CI] 0.19 to 0.84; *P*=0.002) [17].

Further research is needed to determine optimal dosing and long-term outcomes of cardiorespiratory training. From the 14 reviews found, cardiorespiratory exercise is effective for improving movement and health-related quality of life of individuals who have had a stroke [3,8,22].

### Strengthening Interventions

Therapeutic exercise increases strength and activity [9,23], but the specific movement outcomes associated with exercise are unknown. Evidence syntheses from 23 reviews report the following benefits of exercise or strengthening interventions: (1) increased strength [23-25], (2) increased motor activity [23,24], (3) improved balance [26], (4) longer walking distance [15,27], and (5) faster walking speed [25,27-29]. Research suggests that circuit class training can improve walking distance [30], walking speed, and walking ability [31], even for individuals with chronic stroke [30]. However, passive interventions appear less effective; one Cochrane review found little positive evidence for stretching, passive exercises and mobilization of the hemiplegic arm after stroke [32].

Based upon ten systematic reviews, progressive strengthening exercises are effective in improving leg strength and some aspects of gait performance [21,33]. Studies have shown that lower limb resistance training can improve comfortable gait speed and walking distance [34], as well as functional outcomes and quality of life [25]. However, there is insufficient evidence for lower limbs’ effect on walking and balance [35] and pedaling exercise’s effect on motor function [36]. A recent Cochrane review determined that using resistance training to increase walking speed is not supported by evidence [16]. Clinicians can reassure patients who have had a stroke that strengthening does not increase spasticity [23,24] or pain [24]. Among the four reviews examining the evidence for trunk exercises, two reviews concluded that trunk training exercises and lumbar stabilization exercises can improve trunk movement and dynamic sitting balance [37,38]. Aerobic exercise can improve balance of people with subacute and chronic stroke, whereas multisensory programs are less effective [26]. Moreover, balance training is feasible for people in a 1:1 model in the acute stage of stroke and either 1:1 or group therapy for those in the subacute or chronic stroke phase [39]. Exercise should be performed at least 20-60 minutes, 3-4 times a week, for 6-12 weeks. Evidence suggests that more is not necessarily better in the acute stage; exercising 90 minutes or more per day, 5 times per week may not be therapeutic [39].

Four reviews examined the effectiveness of bilateral and unilateral upper limb strengthening. A review from 2010 deemed the evidence for bilateral training as insufficient, when compared to placebo, no intervention, or usual care [40]. Van Delden and colleagues’ meta-analysis reported that a marginally significant mean difference for perceived upper limb activity performance and quality of movement was found for those receiving unilateral training [41]. Although one review favored bilateral therapy’s effect on upper limb function of adults with chronic stroke [42], the most recent review, examining functional task training, bilateral training with rhythmic auditory cues, and robot-assisted training, concluded that these therapy approaches produced results similar to usual therapy [43]. Thus, while bimanual training may improve proximal control, these benefits are offset by the reduced amount and quality of upper limb use from the participants’ perspective [43]. Perhaps the conscious focus for unilateral training positively influences the participants’ opinion of the affected limb’s activity level and movement quality. Despite these findings, a Cochrane review highlighted the need for high-quality randomized controlled trials (RCT) to examine the effects of strength training [7]. In summary, bilateral training appears to produce results that are comparable to usual therapy, but unilateral training may produce better patient-reported outcomes.

### Task-Oriented Training

Task-oriented training (ie, repetitive functional task training, task specific training) is a cost- effective intervention for individuals who have experienced a stroke [44]. Characterized by a composition of 15 components (eg, goal-directed, functional, client centered, repetitious, context specific, progressive, and distributed practice), task-oriented training can be successfully applied when factors of intensity, duration of training, and the proper combination of specific components are incorporated [45]. Among these components, the use of “Distributed practice” and “Feedback” were associated with the largest postintervention effect sizes, and “Random practice” and “Use of clear functional goals” were associated with the largest follow-up effect sizes [45]. Interestingly, the number of components used during an intervention did not correlate with the posttreatment effect size. 

Task-oriented training can improve gait, and can benefit people with chronic stroke [30,46]. Despite States and colleagues’ review concluding insufficient evidence for overground gait training [47], several systematic reviews support intensive repetitive task training’s effects on gait and gait-related activities [22,48,49]. While repetitive task training can produce significant gains in the movement performance of legs (eg, gait velocity, gait endurance, balance, Timed Up and Go Test), such effects do not extend to arm functioning [46,50]. One review suggested that task-oriented training may even be more effective than traditional therapies [51].

To improve aspects of walking ability, treadmill training can increase walking distance and maximum walking speed [52,53]. With regard to supporting body weight or not during treadmill training, it depends upon individuals’ walking ability. A recent Cochrane review found that individuals poststroke *who can walk* benefit more from body-weight supported treadmill training than people with stroke who aren’t able to walk, especially in walking endurance [54]. Veerbeek and colleagues’ review concurred with the use of body-weight supported treadmill training for improving walking distance and noted that electromechanical-assisted gait training with functional electrostimulation can improve maximum gait speed for dependent walkers in the early phase of stroke rehabilitation [21]. Generally, from the 21 reviews focused on walking, people with stroke can increase their walking speed and walking distance by treadmill training and body-weight supported treadmill training [21]. 

Task-oriented training’s effects on performance of daily activities appear to be minimal [49,52] or mixed [49,55,56]. For example, the effects of exercise on activities of daily living (ADL) were mixed; three meta-analyses reported a positive small to medium treatment effect on ADL [21,28,29], but another meta-analysis [57] and systematic review [18] did not find evidence for a favorable effect on ADL. High-intensity of practice results in improved quality of life and, as expected, leisure therapy improves leisure participation [21].

Outcomes from task-oriented training depend upon dosage and intensity (ie, dose x time). Evidence suggests that a higher dosage of task-oriented practice can improve arm functioning [10] and gait speed [21]. Jeon et al suggests training daily for at least two weeks for maximum progress [46]. Based upon 35 reviews, task-oriented training produces modest effects in outcomes related to leg functioning, but the evidence for positive effects on arm functioning is minimal [50].

### Constraint-Induced Movement Therapy

We analyzed 26 systematic reviews and meta-analyses about CIMT, which involves restraining the functioning hand and encouraging the active use of the injured hand. Although the preponderance of evidence for CIMT is positive [9,22,58-63], the high intensity of functionally oriented task practice with the affected arm is difficult to implement because the protocol requires participation for over 90% of waking hours [64,65]. Moreover, CIMT is most beneficial for those who have at least 10 degrees of wrist extension, 10 degrees of thumb abduction/extension, and 10 degrees of finger extension in two other fingers [66]. Fortunately, modified-CIMT (mCIMT), with attenuated protocols or without a physical restraint, produces positive outcomes with less intensity [64].

Most evidence suggests that CIMT improves arm motor function [21,67], arm motor activity, [68,69] and movement quality [21] of the affected upper limb, even when the intensity is reduced and the duration is increased [70]. The schedule for modified CIMT (1 hour/day for 3 days/week for 10 weeks) is more feasible to implement [8]. For improved self-care, one review suggests a higher intensity of CIMT (at least 30 hours over 3 weeks) is beneficial [70], whereas another contends low-intensity mCIMT is effective [21]. In terms of quality of life, a majority of studies reported improved ADL [21], mobility, and participation [70,71]. Even more promising, CIMT appears to improve the amount and quality of active arm movements in people with chronic stroke [72].

Despite the positive findings of CIMT, some researchers have expressed some limitations of CIMT. Pulman and colleagues’ meta-analysis did not find significant improvements in ADL, hand function, and strength [73]. Additionally, a recent Cochrane review acknowledged that CIMT results in improved motor function and less motor impairment, but cautions that these small gains do not reduce disability [74]. While two reviews reported that CIMT’s effects (eg, arm motor activity) can be sustained for up to 6 months [9,68], Cochrane reviews have not found evidence for reducing disability several months after the intervention ended [74,75]. Acknowledging that CIMT can be useful in stroke rehabilitation, one review called for more research to determine if CIMT should be implemented as an adjunct therapy or as a replacement of traditional stroke therapy [76].

There are two issues to consider for clinical applications of CIMT. First, a meta-analysis comparing high and low-intensity CIMT in *acute or sub-acute stroke care* found that low-intensity CIMT may produce better movement and functional use of the affected upper limb [77]. While CIMT appears to produce better upper limb functioning than dose-matched interventions [69], only some of the CIMT RCT results produced minimal clinically important differences [78], raising questions about CIMT’s clinical efficacy [79]. More recently, Etoom and colleagues’ meta-analysis concludes that in comparison to other rehabilitation therapies, CIMT confers relatively small gains [80].

#### Mental Practice

In addition to physical rehabilitation interventions, mental practice can also improve movement and functional performance of patients who have had a stroke [9,81,82]. Mental practice demonstrates cortical activation patterns like those seen with actual movement, per functional imaging.^83^ When combined with conventional therapies, mental practice improves recovery of both upper and lower limbs, as well as for reacquiring daily living skills [83]. Most systematic reviews focusing on mental practice to improve upper limb functioning, such as arm-hand activities, were positive [21,22,82,84,85]. Braun and colleagues reported that mental practice produced short-term gains in arm-hand ability as well as performance of activities [86]. However, Machado and colleagues’ [87] meta-analysis did not find mental practice to be an effective adjunct therapy.

Adding mental practice to upper limb rehabilitation can independently increase functional recovery after stroke [85]. Cha and colleagues’ meta-analysis calculated a medium effect size of .51 (95% CI: 0.27 to 0.75) for functional task training with mental practice during occupational and physical therapy in stroke rehabilitation [88]. Overall, evidence from 14 reviews suggests that mental practice is effective when paired with functional task rehabilitation for individuals who have had a stroke.

#### Mirror Therapy

Mirror therapy (MT) uses a strategically placed mirror to provide visual feedback of the unaffected hand’s movements, creating an illusion that the affected hand is moving similarly. A majority of 12 systematic reviews reported positive effects of mirror therapy’s efficacy for upper limb functioning [7,71,89,90], and two reviews found that MT’s outcomes may be maintained for three to six months [9,91]. Only one review examined the effects of MT on lower limb functioning and found that MT is effective [92]. Whether MT can improve performance of activities of daily living is unclear; one review found MT to be effective based upon four studies [90], and another reported mixed results from three studies [85]. Questions about optimal dosage, timing, and application methods for people with varying stroke severity need to be answered in further research [91,93]. Capitalizing on neuroplasticity, mirror therapy appears to be an efficacious intervention for improving upper limb function after stroke, with moderate quality of evidence from a Cochrane review [94].

#### Neuromuscular Electrical Stimulation

From the 54 articles identified, we retained 31 reviews, and most (21) reported positive findings for neuromuscular electrical stimulation (NMES). In conjunction with functional activities, NMES addresses weakness, coordination, or spasticity, to improve function in poststroke population. NMES has a moderate treatment effect on activity when compared to training [95]. Proponents of NMES cite improved spasticity, range of motion [96], strength, and activity performance [97]. From Nascimento and colleagues’ systematic review with meta-analysis, cyclical electrical stimulation increased strength by a standardized mean difference of 0.47 (95% CI 0.26 to 0.68) and this effect was sustained after the intervention with a small to medium effect size [97].

Evidence suggests that NMES can be effective when combined with other modalities for preventing and treating shoulder subluxation early poststroke [8,98,99]. Also, pairing functional electrical stimulation (FES) with an activity appears to be more beneficial than performing that activity alone (moderate effect size); a synergistic effect results when FES is used for improving upper extremity function (large effect size) [33].

NMES can also help improve lower limb motor performance and walking abilities, such as gait speed [100]. The orthotic effect of FES on walking speed was positive, with a pooled improvement of 0.13 m/s (0.07-0.2) or 38% (22.18-53.8) [101], and this effect has also been demonstrated for individuals with chronic stroke [102]. A recent systematic review examining the carryover effects of lower limb FES to motor performance concluded that FES produced therapeutic effects at the body function and activity levels, but there’s insufficient evidence to ascertain the superiority of FES when compared to matched therapies [103].

Electromyogram (EMG)-triggered electrical stimulation has been shown to have mixed results. Chae asserted that repetitive movement training through transcutaneous cyclic and EMG-triggered NMES could improve stroke survivors’ motor skills [104]. Meilink and colleagues’ systematic review did not find statistically significant difference between EMG-NMES and usual care, citing a lack of rigor with the sampled studies, including non-randomization, small sample size, lack of blinding, and poor contrast to controls [105].

Evidence appraisers cite insufficient evidence for NMES’ efficacy and low-quality trials [7,106-108]. A wide variety of therapy protocols, including duration of therapy [109], as well as heterogeneous samples, contribute to the difficulty in interpreting the evidence. For clinical applications, therapy practitioners should keep in mind that the evidence for NMES for individuals with chronic stroke is insufficient [99]. With more rigorous studies, NMES has the potential to improve functional motor abilities, especially in the acute phase of stroke recovery. Instead of offering FES to all poststroke, guidelines suggest considering electrical stimulation on a trial basis within the first two months poststroke to individuals who demonstrate muscle contraction but cannot move their limbs against resistance [8,110]. Overall, the evidence for FES to improve motor abilities is mixed, and therefore FES is a promising intervention.

## Discussion

### Principal Findings

No single intervention is superior to another in stroke rehabilitation to improve functional performance [7]. The following common movement-focused poststroke interventions have moderate evidence of effectiveness: cardiorespiratory training, therapeutic exercise, task-oriented training (task-specific training), CIMT, mental practice, and mirror therapy. While there are many systematic reviews and meta-analyses about movement interventions, the heterogeneity in samples (eg, acute vs. chronic), interventions (eg, timing, dosage, type), and outcome measures makes analysis of the findings challenging. Combinations of evidence-based interventions across the postacute care continuum to address stroke patients’ motor goals are considered standard care.

NMES is a promising intervention and more rigorous studies are needed to determine its effectiveness, particularly in the acute and subacute phases of stroke rehabilitation. Other interventions associated with stroke rehabilitation (eg, robotic therapy, aquatic therapy, and virtual reality and video gaming) are acquiring more evidence and some (eg, virtual reality) appear to be promising interventions.

To achieve the most recovery of activities of daily living, rehabilitation should be implemented early [7], and continuously across transitions and settings. Usual therapy approaches, including strengthening and functional activities, improved short- and long-distance walking, and thus strengthening interventions appear to be effective, even for individuals with chronic stroke [21,111]. Strengthening interventions may increase muscle functioning but may not necessarily translate to improved performance in ADLs, such as bathing, dressing, and meal preparation.

Recovery from stroke is a dynamic process and therefore rehabilitation professionals *craft* individualized therapy plans to maximize functional performance and participation. Highly effective therapy practitioners understand there is an *art and science* to providing therapy services and achieving excellent patient outcomes [112]. For example, a practitioner may recognize the science aspect by choosing low-intensity CIMT in acute or sub-acute stroke to improve functioning of the weaker arm [77], and use therapeutic rapport or humor to engage and motivate individuals with stroke to perform therapeutic tasks. Knowing when and how to deliver the most effective therapies, coupled with the collaborative and motivating aspect of therapy, will help therapists improve the movement outcomes and quality of care in stroke rehabilitation.

In patient-centered stroke rehabilitation, therapists and patients discuss goals, preferences, and concerns before co-creating treatment plans. Currently, therapists utilize a variety of interventions, considering the evaluation results, and patient/caregiver’s concerns and aims. Consequently, rehabilitation professionals can best serve their clients with stroke by: (1) determining clients’ goals and preferences, (2) thoroughly assessing their capacities and skills, and (3) selecting the interventions with the most relevant evidence that will enable clients to reach their goals.

In addition to the specific therapeutic interventions provided at different levels of care, other factors that influence stroke outcomes include: dosage and intensity of interventions, community support, education of staff and family, and caregiver competency. With regard to dosage, previous reviews have provided limited support for the assumption that a higher dose of exercise-based therapy improves motor recovery after stroke [28,113]. Generally, a dose of 30 to 60 minutes of therapy for five to seven days a week is optimal to improve performance [7,21]. Recently Veerbeek et al asserted that a higher intensity of practice (ie, 17 hours over 10 weeks) results in better outcomes at the body function level, as well as the activities and participation level of the International Classification of Functioning (ICF) [21].

If only one intervention approach is being used for all therapy sessions or if the interventions cannot be named and explained by therapists, patients should seek additional information from their therapy practitioners. Sample questions patients can ask include: (1) Which intervention approaches are you using to achieve my goals? (2) What is the evidence for these approaches? (3) What can I do to maximize my recovery and motor outcomes?

Many patients poststroke may wonder if there is anything they can do at home to improve their motor outcomes. Coupar and colleagues’ Cochrane review asserted that there’s insufficient evidence to form any recommendations about home-based therapy programs to improve arm recovery [114], but recent reviews have cited positive effects of home-based therapies on functional performance [115,116]. Emerging technologies like virtual reality, robotics, and interactive video gaming hold great promise for increasing the dosage of movement-based therapies at home [116,117], which could potentially increase functional outcomes while containing costs.

### Limitations

This evidence review did not include non-English systematic reviews and meta-analyses. We limited our search to the following databases: Medline Complete, PubMed, CINAHL Complete, Cochrane Database, Rehabilitation and Sports Medicine Source, Dissertation Abstracts International, and National Guideline Clearinghouse. Due to our focus on adults’ functional movement of limbs and trunk, we excluded evidence that pertained to pediatrics and speech/swallowing. This review did not include robotic therapy, aquatic therapy, and virtual reality, because they are not commonly implemented in postacute care settings with most patients who have had a stroke. Some interventions lack the efficacy to improve activity performance (eg, neurodevelopmental treatment or NDT) but may be effective for other outcomes, such as improving muscle strength of the arm [9,21,118]. Lastly, we only included Level 1 published evidence and thus there is the possibility of recent RCTs not being included because of publication timelines.

### Future Research

Practice guidelines recommend that stroke patients receive a minimal dose of active practice (ie, one hour each of physical therapy and occupational therapy) per day, at least 5 days per week [119]. Research is needed to identify not only the most effective combinations of movement-based interventions to deliver, but also the best critical window of time to deliver them. We need more research like Kwakkel and colleagues’ meta-analysis, which reported that additional exercise therapy should exceed 16 hours within the first 6 months after stroke to achieve a statistically significant difference in ADL [29]. Such studies offer a clearer picture of the dose of an intervention, timing, and the anticipated functional outcome.

Moreover, therapy protocols need to be researched to increase our understanding of which subgroups benefit the most from certain interventions. In this era of personalized medicine, there may be subsets of stroke survivors who would benefit from a certain therapeutic cocktail of interventions across settings to achieve maximum functional recovery. Multi-site studies and registries could help add to existing databases by collecting data about demographic variables, stroke types, costs, and functional movement outcomes.

Another issue is the wide variation of outcome measures used to measure functional movement. Informed discussions between researchers and clinicians could not only stimulate and focus rehabilitation research, but also pave the path towards attaining consensus about best outcome measures and intervention methods for stroke survivors. Consensus about outcome measures and which interventions to study during the phases of stroke recovery could facilitate comparative effectiveness research.

Additionally, high-quality RCTs are needed to determine if poststroke interventions targeting body functions lead to improved activity and participation [120]. Finally, we need rigorous longitudinal studies to examine cost-effectiveness and the effects of strength training on activity and participation, and to determine to what extent any gains are sustained.

### Conclusions

Patients and rehabilitation professionals may be more reassured that the following interventions possess moderate evidence of effectiveness: cardiorespiratory training, therapeutic exercise, task-oriented training, constraint-induced movement therapy, mental practice, and mirror therapy. More research is needed to determine the optimal timing and dosages of motor interventions, as well as the effectiveness of neuromuscular electrical stimulation. Movement outcomes are influenced by many variables, such as stroke characteristics, intensity, social support, as well as patients’ preferences and goals.

## Abbreviations

ADL: activities of daily living
CIMT: constraint-induced movement therapy
EMG: electromyogram
FES: functional electrical stimulation
ICF: International Classification of Functioning
mCIMT: modified-CIMT
MT: mirror therapy
NMES: neuromuscular electrical stimulation
RCT: randomized controlled trial
SMD: standardized mean difference
TUG: Timed Up and Go

## References

[1] Mozaffarian D, Benjamin EJ, Go AS, Arnett DK, Blaha MJ, Cushman M, Das SR, de Ferranti S, Després JP, Fullerton HJ, Howard VJ, Huffman MD, Isasi CR, Jiménez MC, Judd SE, Kissela BM, Lichtman JH, Lisabeth LD, Liu S, Mackey RH, Magid DJ, McGuire DK, Mohler ER, Moy CS, Muntner P, Mussolino ME, Nasir K, Neumar RW, Nichol G, Palaniappan L, Pandey DK, Reeves MJ, Rodriguez CJ, Rosamond W, Sorlie PD, Stein J, Towfighi A, Turan TN, Virani SS, Woo D, Yeh RW, Turner MB, Writing Group Members, American Heart Association Statistics Committee, Stroke Statistics Subcommittee (2016). Heart Disease and Stroke Statistics-2016 Update: A Report From the American Heart Association. Circulation.

[2] Tariah HA, Hersch G, Ostwald SK (2009). Factors Associated with Quality of Life: Perspectives of Stroke Survivors. Physical & Occupational Therapy In Geriatrics.

[3] Smidt N, de Vet Henrica C W, Bouter LM, Dekker J, Arendzen Johan H, de Bie Rob A, Bierma-Zeinstra Sita M A, Helders Paul J M, Keus Samyra H J, Kwakkel Gert, Lenssen Ton, Oostendorp Rob A B, Ostelo Raymond W J G, Reijman Max, Terwee Caroline B, Theunissen Carlo, Thomas Siep, van Baar Margriet E, van 't Hul Alex, van Peppen Roland P S, Verhagen Arianne, van der Windt Daniëlle A W M, Exercise Therapy Group (2005). Effectiveness of exercise therapy: a best-evidence summary of systematic reviews. Aust J Physiother.

[4] Greenhalgh T, Howick J, Maskrey N, Evidence Based Medicine Renaissance Group (2014). Evidence based medicine: a movement in crisis?. BMJ.

[5] Epstein RM, Street RL (2011). The values and value of patient-centered care. Ann Fam Med.

[6] Khangura S, Konnyu K, Cushman R, Grimshaw J, Moher D (2012). Evidence summaries: the evolution of a rapid review approach. Syst Rev.

[7] Pollock A, Baer G, Campbell P, Choo PL, Forster A, Morris J, Pomeroy VM, Langhorne P (2014). Physical rehabilitation approaches for the recovery of function and mobility following stroke. Cochrane Database Syst Rev.

[8] Winstein CJ, Stein J, Arena R, Bates B, Cherney LR, Cramer SC, Deruyter F, Eng JJ, Fisher B, Harvey RL, Lang CE, MacKay-Lyons M, Ottenbacher KJ, Pugh S, Reeves MJ, Richards LG, Stiers W, Zorowitz RD, American Heart Association Stroke Council‚ Council on CardiovascularStroke Nursing‚ Council on Clinical Cardiology‚Council on Quality of CareOutcomes Research (2016). Guidelines for Adult Stroke Rehabilitation and Recovery: A Guideline for Healthcare Professionals From the American Heart Association/American Stroke Association. Stroke.

[9] Nilsen DM, Gillen G, Geller D, Hreha K, Osei E, Saleem GT (2015). Effectiveness of interventions to improve occupational performance of people with motor impairments after stroke: an evidence-based review. Am J Occup Ther.

[10] Bosch J, O’Donnell MJ, Barreca S, Thabane L, Wishart L (2014). Does Task-Oriented Practice Improve Upper Extremity Motor Recovery after Stroke? A Systematic Review. ISRN Stroke.

[11] Kendall BJ, Gothe NP (2016). Effect of Aerobic Exercise Interventions on Mobility among Stroke Patients. American Journal of Physical Medicine & Rehabilitation.

[12] Pang MYC, Charlesworth SA, Lau RWK, Chung RCK (2013). Using aerobic exercise to improve health outcomes and quality of life in stroke: evidence-based exercise prescription recommendations. Cerebrovasc Dis.

[13] Brazzelli M, Saunders DH, Greig CA, Mead GE (2012). Physical fitness training for patients with stroke: updated review. Stroke.

[14] Brazzelli M, Saunders DH, Greig CA, Mead GE (2011). Physical fitness training for stroke patients. Cochrane Database Syst Rev.

[15] Stoller O, de Bruin ED, Knols RH, Hunt KJ (2012). Effects of cardiovascular exercise early after stroke: systematic review and meta-analysis. BMC Neurol.

[16] Saunders DH, Sanderson M, Hayes S, Kilrane M, Greig CA, Brazzelli M, Mead GE (2016). Physical fitness training for stroke patients. Cochrane Database Syst Rev.

[17] Saunders DH, Sanderson M, Brazzelli M, Greig CA, Mead GE (2013). Physical fitness training for stroke patients. Cochrane Database Syst Rev.

[18] Meek C, Pollock A, Potter J, Langhorne P (2003). A systematic review of exercise trials post stroke. Clin Rehabil.

[19] Saunders DH, Sanderson M, Hayes S, Kilrane M, Greig CA, Brazzelli M, Mead GE (2016). Physical fitness training for stroke patients. Cochrane Database Syst Rev.

[20] Chen MD, Rimmer JH (2011). Effects of exercise on quality of life in stroke survivors: a meta-analysis. Stroke.

[21] Veerbeek J, van Wegen WE, van Peppen PR, van der Wees PJ, Hendriks E, Rietberg M, Kwakkel G (2014). What is the evidence for physical therapy poststroke? A systematic review and meta-analysis. PLoS One.

[22] Langhorne P, Coupar F, Pollock A (2009). Motor recovery after stroke: a systematic review. Lancet Neurol.

[23] Ada L, Dorsch S, Canning C (2006). Strengthening interventions increase strength and improve activity after stroke: a systematic review. Aust J Physiother.

[24] Harris J, Eng J (2010). Strength training improves upper-limb function in individuals with stroke: a meta-analysis. Stroke.

[25] Pak S, Patten C (2008). Strengthening to promote functional recovery poststroke: an evidence-based review. Top Stroke Rehabil.

[26] An M, Shaughnessy M (2011). The effects of exercise-based rehabilitation on balance and gait for stroke patients: a systematic review. J Neurosci Nurs.

[27] van de Port IGL, Wood-Dauphinee S, Lindeman E, Kwakkel G (2007). Effects of exercise training programs on walking competency after stroke: a systematic review. Am J Phys Med Rehabil.

[28] Veerbeek J, Koolstra M, Ket J, van WE, Kwakkel G (2011). Effects of augmented exercise therapy on outcome of gait and gait-related activities in the first 6 months after stroke: a meta-analysis. Stroke.

[29] Kwakkel G, van Peppen R, Wagenaar R, Wood-Dauphinee S, Richards C, Ashburn A, Miller K, Lincoln N, Partridge C, Wellwood I, Langhorne P (2004). Effects of augmented exercise therapy time after stroke: a meta-analysis. Stroke.

[30] Wevers L, van DPI, Vermue M, Mead G, Kwakkel G (2009). Effects of task-oriented circuit class training on walking competency after stroke: a systematic review. Stroke.

[31] English C, Hillier S (2011). Circuit class therapy for improving mobility after stroke: a systematic review. J Rehabil Med.

[32] Winter J, Hunter S, Sim J, Crome P (2011). Hands-on therapy interventions for upper limb motor dysfunction following stroke. Cochrane Database Syst Rev.

[33] Nascimento L, Resende R, Polese J, Magalhães F, Teixeira-Salmela L (2010). Evidences on the effect of strengthening exercises on motor and functional performance of chronic stroke subjects: a systematic review. Revista Terapia Manual.

[34] Mehta S, Pereira S, Viana R, Mays R, McIntyre A, Janzen S, Teasell RW (2012). Resistance training for gait speed and total distance walked during the chronic stage of stroke: a meta-analysis. Top Stroke Rehabil.

[35] Wist S, Clivaz J, Sattelmayer M (2016). Muscle strengthening for hemiparesis after stroke: A meta-analysis. Ann Phys Rehabil Med.

[36] Hancock N, Shepstone L, Winterbotham W, Pomeroy V (2012). Effects of lower limb reciprocal pedalling exercise on motor function after stroke: a systematic review of randomized and nonrandomized studies. Int J Stroke.

[37] Cabanas-Valdés R, Cuchi G, Bagur-Calafat C (2013). Trunk training exercises approaches for improving trunk performance and functional sitting balance in patients with stroke: a systematic review. NeuroRehabilitation.

[38] Ko Dae-Sik, Jung Dae-In, Bae Sang-Yeol (2014). Effect of lumbar stabilization exercises on the balance ability of patients with stroke: a systematic review. J Phys Ther Sci.

[39] Lubetzky-Vilnai A, Kartin D (2010). The effect of balance training on balance performance in individuals poststroke: a systematic review. J Neurol Phys Ther.

[40] Coupar F, Pollock A, van WF, Morris J, Langhorne P (2010). Simultaneous bilateral training for improving arm function after stroke. Cochrane Database Syst Rev.

[41] van Delden AL, Peper CL, Kwakkel G, Beek PJ (2012). A systematic review of bilateral upper limb training devices for poststroke rehabilitation. Stroke Res Treat.

[42] Latimer CP, Keeling J, Lin B, Henderson M, Hale LA (2010). The impact of bilateral therapy on upper limb function after chronic stroke: a systematic review. Disabil Rehabil.

[43] Wolf A, Scheiderer R, Napolitan N, Belden C, Shaub L, Whitford M (2014). Efficacy and task structure of bimanual training post stroke: a systematic review. Top Stroke Rehabil.

[44] French B, Leathley M, Sutton C, McAdam J, Thomas L, Forster A, Langhorne P, Price C, Walker A, Watkins C (2008). A systematic review of repetitive functional task practice with modelling of resource use, costs and effectiveness. Health Technol Assess.

[45] Timmermans AA, Spooren A, Kingma H, Seelen HA (2010). Influence of task-oriented training content on skilled arm-hand performance in stroke: a systematic review. Neurorehabil Neural Repair.

[46] Jeon BJ, Kim WH, Park EY (2015). Effect of task-oriented training for people with stroke: a meta-analysis focused on repetitive or circuit training. Top Stroke Rehabil.

[47] States RA, Pappas E, Salem Y (2009). Overground physical therapy gait training for chronic stroke patients with mobility deficits. Cochrane Database Syst Rev.

[48] French B, Thomas LH, Leathley MJ, Sutton CJ, McAdam J, Forster A, Langhorne P, Price CI, Walker A, Watkins CL (2007). Repetitive task training for improving functional ability after stroke. Cochrane Database Syst Rev.

[49] Eng JJ, Tang PF (2007). Gait training strategies to optimize walking ability in people with stroke: a synthesis of the evidence. Expert Rev Neurother.

[50] French B, Thomas L, Leathley M, Sutton C, McAdam J, Forster A, Langhorne P, Price C, Walker A, Watkins C (2010). Does repetitive task training improve functional activity after stroke? A Cochrane systematic review and meta-analysis. J Rehabil Med.

[51] Rensink M, Schuurmans M, Lindeman E, Hafsteinsdóttir T (2009). Task-oriented training in rehabilitation after stroke: systematic review. J Adv Nurs.

[52] Brogårdh C, Lexell J (2012). Effects of cardiorespiratory fitness and muscle-resistance training after stroke. PM R.

[53] Polese JC, Ada L, Dean CM, Nascimento LR, Teixeira-Salmela LF (2013). Treadmill training is effective for ambulatory adults with stroke: a systematic review. J Physiother.

[54] Mehrholz J, Pohl M, Elsner B (2014). Treadmill training and body weight support for walking after stroke. Cochrane Database Syst Rev.

[55] Gopaul U, van Vliet P (2015). Progressive resistance training improves muscle strength of paretic lower limb in chronic stroke patients: a systematic review. Physiotherapy.

[56] Morris SL, Dodd KJ, Morris ME (2004). Outcomes of progressive resistance strength training following stroke: a systematic review. Clin Rehabil.

[57] van de Port IG, Wood-Dauphinee S, Lindeman E, Kwakkel G (2007). Effects of exercise training programs on walking competency after stroke: a systematic review. Am J Phys Med Rehabil.

[58] Fleet A, Page SJ, MacKay-Lyons M, Boe SG (2014). Modified constraint-induced movement therapy for upper extremity recovery post stroke: what is the evidence?. Top Stroke Rehabil.

[59] Hakkennes S, Keating JL (2005). Constraint-induced movement therapy following stroke: a systematic review of randomised controlled trials. Aust J Physiother.

[60] Medical Advisory Secretariat‚ Health Quality Ontario (2011). Constraint-induced movement therapy for rehabilitation of arm dysfunction after stroke in adults: an evidence-based analysis. Ont Health Technol Assess Ser.

[61] Bjorklund A, Fecht A (2006). The effectiveness of constraint-induced therapy as a stroke intervention: a meta-analysis. Occup Ther Health Care.

[62] Wolf SL, Blanton S, Baer H, Breshears J, Butler AJ (2002). Repetitive task practice: a critical review of constraint-induced movement therapy in stroke. Neurologist.

[63] Mark VW, Taub E (2004). Constraint-induced movement therapy for chronic stroke hemiparesis and other disabilities. Restor Neurol Neurosci.

[64] Shi YX, Tian JH, Yang KH, Zhao Y (2011). Modified constraint-induced movement therapy versus traditional rehabilitation in patients with upper-extremity dysfunction after stroke: a systematic review and meta-analysis. Arch Phys Med Rehabil.

[65] Reiss AP, Wolf SL, Hammel EA, McLeod EL, Williams EA (2012). Constraint-Induced Movement Therapy (CIMT): Current Perspectives and Future Directions. Stroke Res Treat.

[66] Blanton S, Wilsey H, Wolf SL (2008). Constraint-induced movement therapy in stroke rehabilitation: perspectives on future clinical applications. NeuroRehabilitation.

[67] Corbetta D, Sirtori V, Moja L, Gatti R (2010). Constraint-induced movement therapy in stroke patients: systematic review and meta-analysis. Eur J Phys Rehabil Med.

[68] Thrane G, Friborg O, Anke A, Indredavik B (2014). A meta-analysis of constraint-induced movement therapy after stroke. J Rehabil Med.

[69] Stevenson T, Thalman L, Christie H, Poluha W (2012). Constraint-Induced Movement Therapy Compared to Dose-Matched Interventions for Upper-Limb Dysfunction in Adult Survivors of Stroke: A Systematic Review with Meta-analysis. Physiother Can.

[70] Peurala SH, Kantanen MP, Sjögren T, Paltamaa J, Karhula M, Heinonen A (2012). Effectiveness of constraint-induced movement therapy on activity and participation after stroke: a systematic review and meta-analysis of randomized controlled trials. Clin Rehabil.

[71] Pulman J, Buckley E (2013). Assessing the efficacy of different upper limb hemiparesis interventions on improving health-related quality of life in stroke patients: a systematic review. Top Stroke Rehabil.

[72] McIntyre A, Viana R, Janzen S, Mehta S, Pereira S, Teasell R (2012). Systematic review and meta-analysis of constraint-induced movement therapy in the hemiparetic upper extremity more than six months post stroke. Top Stroke Rehabil.

[73] Pulman J, Buckley E, Clark-Carter D (2013). A meta-analysis evaluating the effectiveness of two different upper limb hemiparesis interventions on improving health-related quality of life following stroke. Top Stroke Rehabil.

[74] Corbetta D, Sirtori V, Castellini G, Moja L, Gatti R (2015). Constraint-induced movement therapy for upper extremities in people with stroke. Cochrane Database Syst Rev.

[75] Sirtori V, Corbetta D, Moja L, Gatti R (2009). Constraint-induced movement therapy for upper extremities in stroke patients. Cochrane Database Syst Rev.

[76] Kelly A, Blackwell A, Helms-Jaye S, Cheek T, Collins K, Dolbow DR (2014). The effects of constraint-induced movement therapy post-stroke. Clinical Kinesiology (Online Edition).

[77] Nijland R, Kwakkel G, Bakers J, van Wegen E (2011). Constraint-induced movement therapy for the upper paretic limb in acute or sub-acute stroke: a systematic review. Int J Stroke.

[78] Bonaiuti D, Rebasti L, Sioli P (2007). The constraint induced movement therapy: a systematic review of randomised controlled trials on the adult stroke patients. Eura Medicophys.

[79] Castellini G, Gianola S, Banzi R, Corbetta D, Gatti R, Sirtori V, Gluud C, Moja L (2014). Constraint-induced movement therapy: trial sequential analysis applied to Cochrane collaboration systematic review results. Trials.

[80] Etoom M, Hawamdeh M, Hawamdeh Z, Alwardat M, Giordani L, Bacciu S, Scarpini C, Foti C (2016). Constraint-induced movement therapy as a rehabilitation intervention for upper extremity in stroke patients: systematic review and meta-analysis. Int J Rehabil Res.

[81] El-Shennawy SA, El-Wishy AA (2012). A systematic review of efficacy of mental practice in chronic stroke rehabilitation. Egyptian Journal of Neurology, Psychiatry & Neurosurgery.

[82] Nilsen DM, Gillen G, Gordon AM (2010). Use of mental practice to improve upper-limb recovery after stroke: a systematic review. Am J Occup Ther.

[83] García Carrasco D, Aboitiz CJ (2016). Effectiveness of motor imagery or mental practice in functional recovery after stroke: a systematic review. Neurologia.

[84] Calayan LSM, Dizon JMR (2009). A systematic review on the effectiveness of mental practice with motor imagery in the neurologic rehabilitation of stroke patients. Internet Journal of Allied Health Sciences & Practice.

[85] Hayward KS, Barker RN, Carson RG, Brauer SG (2014). The effect of altering a single component of a rehabilitation programme on the functional recovery of stroke patients: a systematic review and meta-analysis. Clin Rehabil.

[86] Braun S, Kleynen M, van Heel T, Kruithof N, Wade D, Beurskens A (2013). The effects of mental practice in neurological rehabilitation; a systematic review and meta-analysis. Front Hum Neurosci.

[87] Machado S, Lattari E, de Sa AS, Rocha NF, Yuan TF, Paes F, Wegner M, Budde H, Nardi AE, Arias-Carrión O (2015). Is mental practice an effective adjunct therapeutic strategy for upper limb motor restoration after stroke? A systematic review and meta- analysis. CNS Neurol Disord Drug Targets.

[88] Cha YJ, Yoo EY, Jung MY, Park SH, Park JH (2012). Effects of functional task training with mental practice in stroke: a meta analysis. NeuroRehabilitation.

[89] Ezendam D, Bongers RM, Jannink MJ (2009). Systematic review of the effectiveness of mirror therapy in upper extremity function. Disabil Rehabil.

[90] Thieme H, Mehrholz J, Pohl M, Behrens J, Dohle C (2013). Mirror therapy for improving motor function after stroke. Stroke.

[91] Mei Toh SF, Fong KNK (2012). Systematic Review on the Effectiveness of Mirror Therapy in Training Upper Limb Hemiparesis after Stroke. Hong Kong Journal of Occupational Therapy.

[92] Hung GKN, Li CTL, Yiu AM, Fong KNK (2015). Systematic Review: Effectiveness of Mirror Therapy for Lower Extremity Post-Stroke. Hong Kong Journal of Occupational Therapy.

[93] Rothgangel AS, Braun SM, Beurskens AJ, Seitz RJ, Wade DT (2011). The clinical aspects of mirror therapy in rehabilitation: a systematic review of the literature. Int J Rehabil Res.

[94] Pollock A, Farmer SE, Brady MC, Langhorne P, Mead GE, Mehrholz J, van Wijck F (2014). Interventions for improving upper limb function after stroke. Cochrane Database Syst Rev.

[95] Howlett OA, Lannin NA, Ada L, McKinstry C (2015). Functional electrical stimulation improves activity after stroke: a systematic review with meta-analysis. Arch Phys Med Rehabil.

[96] Stein C, Fritsch CG, Robinson C, Sbruzzi G, Plentz RD (2015). Effects of Electrical Stimulation in Spastic Muscles After Stroke: Systematic Review and Meta-Analysis of Randomized Controlled Trials. Stroke.

[97] Nascimento LR, Michaelsen SM, Ada L, Polese JC, Teixeira-Salmela LF (2014). Cyclical electrical stimulation increases strength and improves activity after stroke: a systematic review. J Physiother.

[98] Vafadar AK, Côté JN, Archambault PS (2015). Effectiveness of functional electrical stimulation in improving clinical outcomes in the upper arm following stroke: a systematic review and meta-analysis. Biomed Res Int.

[99] Ada L, Foongchomcheay A (2002). Efficacy of electrical stimulation in preventing or reducing subluxation of the shoulder after stroke: a meta-analysis. Aust J Physiother.

[100] Robbins SM, Houghton PE, Woodbury MG, Brown JL (2006). The therapeutic effect of functional and transcutaneous electric stimulation on improving gait speed in stroke patients: a meta-analysis. Arch Phys Med Rehabil.

[101] Kottink AI, Oostendorp LJ, Buurke JH, Nene AV, Hermens HJ, IJzerman MJ (2004). The orthotic effect of functional electrical stimulation on the improvement of walking in stroke patients with a dropped foot: a systematic review. Artif Organs.

[102] Roche A, Laighin GO, Coote S (2009). Surface-applied functional electrical stimulation for orthotic and therapeutic treatment of drop-foot after stroke ? a systematic review. Physical Therapy Reviews.

[103] Kafri M, Laufer Y (2015). Therapeutic effects of functional electrical stimulation on gait in individuals post-stroke. Ann Biomed Eng.

[104] Chae J (2003). Neuromuscular electrical stimulation for motor relearning in hemiparesis. Phys Med Rehabil Clin N Am.

[105] Meilink A, Hemmen B, Seelen HAM, Kwakkel G (2008). Impact of EMG-triggered neuromuscular stimulation of the wrist and finger extensors of the paretic hand after stroke: a systematic review of the literature. Clin Rehabil.

[106] Pelton T, van Vliet P, Hollands K (2012). Interventions for improving coordination of reach to grasp following stroke: a systematic review. Int J Evid Based Healthc.

[107] Quandt F, Hummel FC (2014). The influence of functional electrical stimulation on hand motor recovery in stroke patients: a review. Exp Transl Stroke Med.

[108] Hayward K, Barker R, Brauer S (2010). Interventions to promote upper limb recovery in stroke survivors with severe paresis: a systematic review. Disabil Rehabil.

[109] Bolton DA, Cauraugh JH, Hausenblas HA (2004). Electromyogram-triggered neuromuscular stimulation and stroke motor recovery of arm/hand functions: a meta-analysis. J Neurol Sci.

[110] National Clinic Guideline Centre (2013). NICE Clinical Guidelines, No. 162.

[111] Ferrarello F, Baccini M, Rinaldi LA, Cavallini MC, Mossello E, Masotti G, Marchionni N, Di Bari M (2011). Efficacy of physiotherapy interventions late after stroke: a meta-analysis. J Neurol Neurosurg Psychiatry.

[112] Kristensen HK, Persson D, Nygren C, Boll M, Matzen P (2011). Evaluation of evidence within occupational therapy in stroke rehabilitation. Scand J Occup Ther.

[113] Cooke EV, Mares K, Clark A, Tallis RC, Pomeroy VM (2010). The effects of increased dose of exercise-based therapies to enhance motor recovery after stroke: a systematic review and meta-analysis. BMC Med.

[114] Coupar F, Pollock A, Legg LA, Sackley C, van Vliet P (2012). Home-based therapy programmes for upper limb functional recovery following stroke. Cochrane Database Syst Rev.

[115] Siemonsma P, Döpp C, Alpay L, Tak E, Meeteren Nv, Chorus A (2014). Determinants influencing the implementation of home-based stroke rehabilitation: a systematic review. Disabil Rehabil.

[116] Wittmann F, Held JP, Lambercy O, Starkey ML, Curt A, Höver R, Gassert R, Luft AR, Gonzenbach RR (2016). Self-directed arm therapy at home after stroke with a sensor-based virtual reality training system. J Neuroeng Rehabil.

[117] Laver KE, George S, Thomas S, Deutsch JE, Crotty M (2015). Virtual reality for stroke rehabilitation. Cochrane Database Syst Rev.

[118] Langhammer B, Stanghelle JK (2011). Can physiotherapy after stroke based on the Bobath concept result in improved quality of movement compared to the motor relearning programme. Physiother Res Int.

[119] Wright L, Hill KM, Bernhardt J, Lindley R, Ada L, Bajorek BV, Barber PA, Beer C, Golledge J, Gustafsson L, Hersh D, Kenardy J, Perry L, Middleton S, Brauer SG, Nelson MR, National Stroke Foundation Stroke Guidelines Expert Working Group (2012). Stroke management: updated recommendations for treatment along the care continuum. Intern Med J.

[120] Faria-Fortini I, Michaelsen SM, Cassiano JG, Teixeira-Salmela LF (2011). Upper extremity function in stroke subjects: relationships between the international classification of functioning, disability, and health domains. J Hand Ther.

